# Centrifugal Pump Fault Detection with Convolutional Neural Network Transfer Learning

**DOI:** 10.3390/s24082442

**Published:** 2024-04-11

**Authors:** Cem Ekin Sunal, Vladan Velisavljevic, Vladimir Dyo, Barry Newton, Jake Newton

**Affiliations:** 1 School of Computer Science and Technology, University of Bedfordshire, Luton LU1 3JU, UKvladan.velisavljevic@beds.ac.uk (V.V.); 2 Department of Electronic Engineering, Royal Holloway, University of London, Egham TW20 0EX, UK; 3 Uptime Systems Ltd., Leighton Buzzard LU7 4WG, UK; barry.newton@uptime.uk.com (B.N.);

**Keywords:** machine learning, Internet of things, centrifugal pump, condition monitoring

## Abstract

The centrifugal pump is the workhorse of many industrial and domestic applications, such as water supply, wastewater treatment and heating. While modern pumps are reliable, their unexpected failures may jeopardise safety or lead to significant financial losses. Consequently, there is a strong demand for early fault diagnosis, detection and predictive monitoring systems. Most prior work on machine learning-based centrifugal pump fault detection is based on either synthetic data, simulations or data from test rigs in controlled laboratory conditions. In this research, we attempted to detect centrifugal pump faults using data collected from real operational pumps deployed in various places in collaboration with a specialist pump engineering company. The detection was done by the binary classification of visual features of DQ/Concordia patterns with residual networks. Besides using a real dataset, this study employed transfer learning from the image detection domain to systematically solve a real-life problem in the engineering domain. By feeding DQ image data into a popular and high-performance residual network (e.g., ResNet-34), the proposed approach achieved up to 85.51% classification accuracy.

## 1. Introduction

Centrifugal pumps are ubiquitous in industrial and domestic applications and represented a 51% market share of the USD 98.02 billion pump market in 2020 [1]. As with any other mechanical equipment, centrifugal pumps are subject to wear and can develop faults related to bearing, sealing and blockage, in addition to electrical faults within the driving motor. The timely detection of these faults is essential to prevent accidents and disruptions in industrial and domestic processes.

Motor current signature analysis (MCSA) is an established condition-monitoring method based on monitoring distinct electric current patterns of the stator in the induction motor [2], which drives the pump, using a combination of signal processing, statistical techniques and machine learning [3]. The MCSA-based systems can be deployed by simply attaching current clamps to power supply wires, which can be done away from the pump itself. Compared with other sensing modalities, such as vibration, acoustic [4] and pressure, MCSA does not require physical access to the pump, which makes it practical and sometimes the only feasible method for some applications, such as submerged sewage pumps. MCSA has been applied for the detection of broken rotor bars [5,6,7], stator winding faults [8], cavitation faults [9,10], blockages [10,11,12], bearing faults [13], impeller defects [14], shaft wear [15], and inner and outer race faults [16].

Recently, the emergence of advanced machine learning algorithms, in combination with low-cost sensing hardware, has opened up new opportunities for intelligent preventative maintenance. However, many machine learning-based MCSA fault detection works generally relied on simulation or synthetic data [17]. The studies that used MCSA on real physical motor/pump data [17] utilised test rigs in controlled laboratory conditions. Often, the faults would be artificially generated by drilling a hole in a rotor bar to mimic a broken rotor bar condition [18], using polystyrene to clog impellers [19], etc., The prior work on applying machine learning to real pumps that have been deployed in real-world conditions is limited and not well documented. This is often due to the scarcity of pump-related datasets, cost and significant practical difficulties in collecting data from real pumps [17].

The presented study applied a machine learning algorithm to reliably detect motor faults using data obtained from several operational pumps running at various locations in the UK. This paper first presents a distributed IoT system designed to sense, transmit, store and process MCSA data using low-cost hardware developed in this project. It then describes a unique dataset collected from centrifugal pumps deployed in the UK over a period of almost 2 years between 2021 and 2023. The dataset included data from non-faulty operational pumps; pumps with diagnosed faults; and intentionally damaged pumps, including damage such as impeller damage, stator winding faults, excessive vibrations, cavitations and/or bearing damage faults. A novel fault detection method is then proposed based on DQ pattern analysis that built upon work by [11,20,21,22]. The method extracts DQ patterns and feeds them into convolutional neural network transfer learning for analysis to provide the binary classification of different type of faults. The paper makes the following contributions:

- A novel IoT-based system for measuring, collecting and storing MCSA data from operational pumps.
- A novel machine learning method for pump fault detection that uses convolutional neural network transfer learning on DQ patterns.
- An evaluation of the proposed algorithm using custom dataset from operational pumps.
- An experience in practical challenges and limitations in implementing such a system.

To the best of our knowledge, this is the first study that applied machine learning for fault detection using real datasets from operational pumps.

## 2. Related Work

### 2.1. IoT-Based Induction Motor Monitoring

The application of machine learning for the condition monitoring of induction motors is an active research area. A comprehensive analysis and review of machine learning for fault detection in centrifugal pumps is available in [17]. Considering that an inductive motor is an essential component that drives a centrifugal pump, the analysis below includes relevant work on MCSA-based induction motor fault detection. The emergence of advanced machine learning algorithms, in combination with low-cost sensing hardware, has opened up new opportunities for intelligent preventative maintenance. Most prior work on IoT-based induction motor monitoring focuses on operational conditions, electrical efficiency or output power of the motors. With few exceptions, most experimental evaluation has been done in a laboratory test bench. Sen et al. [23] designed a system for induction motor monitoring with current measurements. Similarly to our work, the authors evaluated their system on deployed operational systems, which, in their case, was in a textile factory. However, the work focussed on hardware and software design but did not provide much details about the algorithms or dataset. Cano-Ortego and Sanchez-Sutil [24] designed a low-cost system to acquire the voltage, current and mechanical parameters for improving the energy efficiency and power factor of induction motors. The system protects the motor against overloads and uses LoRa for long range communication and stores data in Google Cloud. The presented prototype was used in a case study using electrical machines in the university laboratory. Carvalho et al. [25] proposed a method for monitoring the efficiency and output power of three-phase induction motors using three-phase voltages, currents and the equivalent circuit of the machine. The system was evaluated on an experimental test bench. Sridhar et al. [26] describes a system for fault classification of induction motors by processing stator current measurements using neural networks. Kunthong et al. [27] implemented an IoT system for monitoring induction motor vibration, current and temperature in electric vehicles.

### 2.2. DQ Pattern Analysis

The presented approach was inspired by the discussion of DQ patterns and related motor conditions by Irfan et al. in [11,20,21], as well as a Park vector application for DQ [22]. Irfan et al. [11] used a three-phased 1.5 hp, 3450 rpm and 60 Hz centrifugal pump and proposed an electric diagnostic technique to detect CP faults without needing extra sensors. They measured the three-phase line currents with the voltage; then, they transformed the data to two-phase DQ patterns. The authors showed that the shapes of the obtained DQ pattern plots were affected by the motor faults, for example, the impeller or blockage faults disturbed the hexagonal DQ plot to a fan-shaped circle. They obtained their dataset of 1000 samples, which were sampled at a 4000 Hz rate by using a PXIe-1082 data acquisition module [11]. Their fault detection attempt was undertaken with pattern classification based on statistical indices after plotting the DQ patterns.

Therefore, it is believed that DQ is an intuitive method for detecting induction motor centrifugal pump faults. Thanks to their distinct characteristics discussed in Section 3.2, it is thought that a smaller-scale state-of-the-art convolutional neural network (CNN) that does not require a high amount of resources and can be deployed to consumer-grade hardware will be satisfactory to solve the detection problem. As CNNs are popular tools for computer vision problems thanks to its computational efficiency and parameter-sharing capability, we think they can be scaled to consumer-grade devices usage to detect simple image pattern recognition problem posed by DQ patterns. Given that their features are not as complex as the real world objects that ImageNet has, we believe that relatively small but proven state-of-the-art residual networks, like ResNet-34, can be used under transfer learning to efficiently solve the problem. Residual networks are the improved version of CNNs, where they use residual layers to keep gradients from vanishing [28] and allow for the development of deeper-layered CNNs.

In order to detect the motor faults in the induction motors or centrifugal pumps, the usage of CNNs were explored by researchers like Valtierra-Rodriguez et al. [7]. They used a CNN to to detect broken rotor bars with a short-time Fourier transform signals map of the current signatures, with a final accuracy as high as 100%. They used a WEG-00136APE48T in-test motor that had two poles, 28 bars and a nominal power of 1 hp, and worked with 220 VAC at 60 Hz. Their dataset, however, only had 400 current signals, with a 3:1 training-to-validation dataset ratio. For more detailed analysis of using images for classification for industrial, health and other applications, we refer the reader to other work, e.g., [29,30,31,32,33].

## 3. Methods

This section presents the methods used in the creation of the model’s pipeline. We first describe the Park transformation, which was used to convert three-phase measurements into two components named D and Q. The visualisation of these two components on a two-dimensional plot produces a circular shape for a healthy motor, whereas any distortions may indicate a faulty condition. The proposed approach uses a convolutional neural network algorithm to capture these distortions to detect faults. It should be noted that the idea of using images for classification purposes have been actively explored in various other domains, e.g., [30,31].

### 3.1. Park Transformation

For the data transformation we decided to employ Park transform. The transform can be used by converting the three-phase motor current signature to a two-phase system that has two components named D and Q in order to describe three-phase IM phenomena with Park’s vector [6]:

(1) $$x_{d}\left(t\right)=\sqrt{\frac{2}{3}}x_{a}\left(t\right)-\frac{1}{\sqrt{6}}\left(x_{b}\left(t\right)-x_{c}\left(t\right)\right)$$

(2) $$x_{q}\left(t\right)=\frac{1}{\sqrt{2}}\left(x_{b}\left(t\right)-x_{c}\left(t\right)\right)$$

The space vector:

(3) $$x_{s}\left(t\right)=\sqrt{x_{d}^{2}\left(t\right)+x_{q}^{2}\left(t\right)}$$

where $x_{d}$ and $x_{q}$ are Park’s vector components. The component of these vectors are derived from the three weighted phases and their subtractions from each other [6]. The Park transform is essentially a conversion of three-phase motor current data to two components called *d* and *q*. DQ/Concordia patterns are the plots of *d* and *q* against each other. They have various (disturbed) shapes that give us an indication whether the sampled motor signature has a failure or not. The components D and Q are then used to generate a 2D image, which is used for fault classification, as described in the following section.

### 3.2. The Identification of DQ Patterns

DQ has several implementation areas in the literature and the ability to detect several motor faults. Given that it can be applied to the three-phase induction motors that any system (e.g., wind turbines) can have, it has the potential for a wide range of usage. Each fault can have distinct DQ patterns. Currently there are only a handful of publications that demonstrate the change in DQ pattern between healthy and faulty, and in this section, they will be discussed. According to the authors of [11,20], the shape of a healthy motor’s DQ data is perfectly circular. Aside from the ability to detect the shape of healthy motors, a DQ pattern is reported to demonstrate the presence of impeller, blockage, BRB and short-circuit turn failures.

BRBs and/or short circuit between turns faults: For every turn, the tracked MCSA plot produces a slightly altered version of every cycle and causes an elliptical shape of the Park transform [21]. The difference can also be seen more clearly between healthy and a BRB fault problem in [20]. The DQ plot does not overlap the area it has already passed due to fault-induced phases.

Impeller faults and pipe blockages: The authors of [11] used DQ plots to illustrate how faults gradually change the shape of the DQ plots. They used a (hand-valve) half-pipe block, full-pipe block, (artificially) damaged impeller surface and 120-degree angled damaged impeller surface. According to their observations in line plots, the healthy shape of a pump is relatively hexagonal. However, this status changes when the aforementioned faults are introduced. They observed that for every increasingly severe fault iteration, the hexagonal shape is lost and a fan shape is constructed. As discussed earlier, this newly formed fan shape is not perfect, and like BRB faults, for every period, the plot does have a slightly different radius and period length. This causes the fan shape to be disrupted and not uniform in parallel to the increasing fault severity.

Therefore, our aim was to not only focus on these faults, but also investigate the affects of other faults on the DQ plot. In the end, we strived to create a fault-blind model that can ideally detect any fault without prior knowledge or at least with limited knowledge.

## 4. Implementation

### 4.1. System Architecture

**Figure 1:**
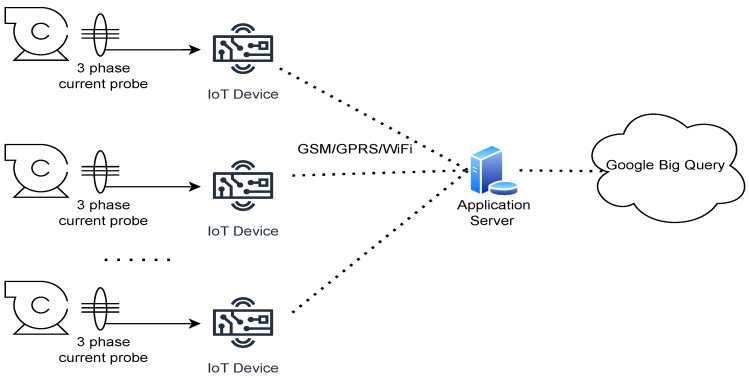
System architecture.

The system consists of custom-developed IoT devices developed by Uptime Systems [34] and deployed at five customer sites, a Google Big Query Database and an application server, as shown in Figure 1. The IoT devices were Arduino-based and used SCT013 20A/1V transducers for AC current sensing. A number of devices were fabricated, deployed and maintained over an almost 2-year period from 2021 to 2023. The IoT devices were deployed near the pump control panels, measured the AC current in three phases and then transmitted the raw data to the application server over a GSM/GPRS link. The measurements were performed on each pump activation, at most every 60 min at 1500 Hz, 3000 Hz and 4500 Hz sampling rates, collecting 1500 samples for each phase per measurement. These sampling rates were consistent with prior work, which ranged from 1500 Hz to 50,000 Hz for detecting various faults, such as broken rotor bar, inter turn and bearing faults. As some devices were deployed in basements or geographical areas with no or poor cellular connectivity, some devices were equipped with Wi-Fi modules to connect to a local WLAN.

Finally, a custom application server was developed in the Flask framework to process incoming data streams; generate alarms; store/retrieve sensor data from the Google Big Query Database; and finally, visualise the data and system status on a dashboard. The description and characteristics of the pumps, as well as their faults, are provided later in the following subsections.

### 4.2. Feature Extraction and Classification

**Figure 2:**
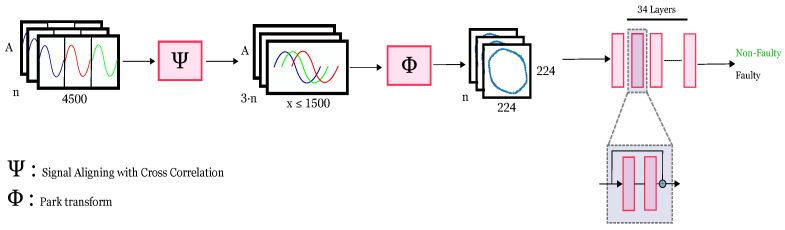
Machine learning pipeline.

The detection process starts with the data retrieval from the database, as shown in the left-most block in Figure 2. Each batch had a dimension of A·4500·n, where *A*, 4500 and *n* mean the amplitude, concatenated three phase data points and the total number of collected signatures, respectively. Given that the collected data for each phase were not aligned in phase due to the hardware limitations, we developed a function ψ to align the three phases. The original data shape had a dimension of 1500·3, where 1500 was the total amount of data points sampled per phase and 3 was the total phase number. The aligning process (ψ) was done via the calculation of the cross-correlation of each signal with respect to each other. Then, the optimization step to find the minimum amount required to shift and cut the signal from their ends to align was performed. All sequences were calculated to minimize the data loss due to alignment (e.g., aligning all phases with respect to the first, second or third phase). The obtained signals were aligned with the dimensions of A·(x≤1500)·(3·n), where *x* was the total amount of data points per phase. As phase alignment can lead to data loss, the actual number of data point for each phase could be less than 1500. The choice of 1500 samples per phase was due to hardware limitations and can be increased in the future.

Next, the DQ components were extracted from the aligned three-phase data using the Park transform (marked as ϕ in Figure 2), resulting in batches of (x≤1500)·(2·n) dimensions, with a *d* and *q* component for every point. The D and Q components were then plotted as a gridless RGB image for further pre-processing by a machine learning model. The pre-processing consisted of resizing, center cropping to an appropriate model input dimension (224·224·3) and normalisation, before finally being fed to the model (e.g., ResNet-34).

The final step was the ML detection through feeding DQ images through consumer-grade hardware and a state-of-the-art residual network. Although the current model results were classified as either “faulty” or “non-faulty”, the final layer can be extended to hold many fault classes, as mentioned in the future work section. One particular benefit of plotting the DQ values and feeding them as images is that the plot will not have axis values. This means that the pumps with higher voltages will not cause any overfitting with their higher DQ values. This can help to eliminate the bias based on the largeness and purely focus on the image features.

### 4.3. Data Processing and Augmentation

The classification labels were set to “faulty/1” and “non-faulty/0”. The training-to-validation ratio was set to ∼3:1 and the faulty-to-non-faulty ratio was set to ∼1:1. The validation and training datasets were prepared with 1500 and 3000 Hz sampling frequencies. The testing dataset consisted of two pumps that were sampled with three different sampling rates (1500, 3000 and 4500 Hz) to provide an additional unseen sampling distribution class to the model’s performance. For each sampling rate, at least 50 signatures were collected to have a balanced dataset. The authors took this approach to create varied distributions of unseen datasets to train robust models. Furthermore, with the varied “unseen” sampling frequency (4500 Hz), the model’s robustness to unseen conditions was also examined. Testing different sampling rates allowed us to not only conduct device reliability testing but also collect data of different distributions from varying conditions. However, evaluating the model performance for different sampling rates is potential future work.

The specifications of the training, validation and testing datasets are presented in Table 1, Table 2 and Table 3, respectively, and illustrate the variations in the gathered dataset. The pumps’ speed and voltage are included to show the variety of data distributions used in training the model, as it might influence the fault development and may create a variety of features for the model to learn. For example, ref. [35] suggested that the speed can be used to understand the pump performance degradation. They also argued that the induction motor condition can be estimated by spectral analysis of the motor current and voltage waveforms. Therefore, it is important to share this information for potential further research areas when there is even more data to independently investigate the effects of these data on fault development. The faults were manually identified by the pump maintenance company engineers based on their knowledge of the pumps.

As noted in our prior work [17], there are very few publications that used real data, and among those publications, the dataset size is pretty small, which presents an issue for developing a robust machine learning model. For this reason, data augmentation techniques were used to increase our dataset sixfold. The total list of these augmentations for the training dataset was vertical mirroring, horizontal mirroring, 90-degree clockwise rotation, 180-degree clockwise rotation and 270-degree clockwise rotation. During the augmentation, the original image was used to obtain five augmented versions, which were saved in the dataset. The dataset sizes can be seen in Table 4. It should be noted that the training dataset did not have any data that were sampled at a 4500 Hz frequency. Due to the scarcity of data, some of the 3000 Hz data from two pump sites were artificially noised. The data were used as part of the training and validation. However, all testing was performed only with data from real pumps.

The booster test pumps’ signatures were never used for training and validation and were only used for the final result reporting and comparison.

## 5. Results and Discussion

### 5.1. Results Comparison

Table 5 shows the precision, recall and accuracy for testing and validation datasets. As a reminder, the data for the validation dataset was collected from real pumps that were different from those used for training. Therefore, the results present the model’s performance against the unseen validation and testing datasets. Table 6 shows that the ResNet-34 model performed the best at a higher sampling frequency (i.e., 4500 Hz) for both faulty and non-faulty pumps, despite not being trained on that frequency. However, it was also observed that non-faulty pumps had comparatively worse accuracy than the faulty pumps.

**Figure 3:**
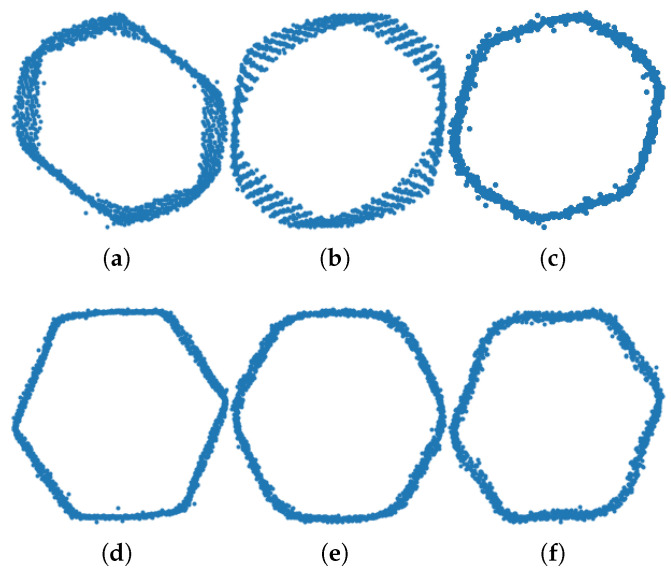
DQ patterns for faulty (**a**–**c**) and non-faulty pumps (**d**–**f**).

For a further demonstration of the model’s performance, the following metrics were used: precision, recall, accuracy and F1-score, as can be seen in Table 5. The datasets’ sampling frequency for these experiments can be found in Section 4.3. These metrics are defined as follows: Precision = TP/(TP + FP), Recall = TP/(TP + FN), Accuracy = (TP + TN)/(TP + TN + FP + FN) and F1-score = 2TP/(2TP + FP + FN). These metrics can demonstrate the model’s robustness against overfitting. With the higher than 80% F1-score, we believe our model was capable enough to produce reliable decisions. Figure 3 shows the DQ patterns for the faulty and non-faulty pumps. It can be seen that the faulty pump signatures used in our experiments were characterised by irregular and distorted shapes compared with the non-faulty pumps. Table 7 shows the confusion matrices for 1500 Hz, 3000 Hz and 4500 Hz data.

### 5.2. Discussion and Lessons Learned

The authors believe that the application of a convolutional neural network model to a non-conventional signal data type is a novel and relatively unique method. Despite its unorthodoxy, the model obtained decent results on both the validation dataset and the testing datasets. Furthermore, its relative performance on varying sampling rates also shows that it is a robust technique. The accuracy seems consistent with the reported fault detection accuracies in prior work [5,6,14], which ranged from 83.2% to 100%.

Although this study focuses on the machine learning aspect, the most significant challenges were faced in building, deploying and maintaining the infrastructure to collect the data. First, collecting faulty signatures from real pumps requires solving not only technical but also business dilemmas. Collecting faulty signatures requires a faulty pump or pump that is going to develop a fault soon, and thus, contradicts the customers’ needs for continuous and non-faulty operation of their systems. The early replacements of these pumps is desirable to ensure smooth operation but can reduce the number of collected faulty signatures. Obtaining a sufficient number of faulty signatures to train the machine learning algorithm to correctly identify and label the faults has indeed been one of the major challenges. This is because modern pumps may operate for many years, and even after developing some faulty condition, may run under workable conditions for a long time. In addition, pumps can have multiple faults at the same time, which may complicate correct labelling and classification. To some extent, this could be mitigated by deploying data loggers on older pumps, which are more likely to fail, based on the maintenance engineer’s knowledge.

A more reliable way to understand whether those motors/pumps are faulty or not is to intentionally damage the motor/pump to create the condition or investigate the pump/motor itself to look out for faults. While intentionally damaging the system is neither profitable nor exactly close to real life conditions, the close-up investigation of the system can render the pump/motor useless and create downtime for working systems. This is why we took extra care to limit such possibilities by using only pumps with known labels (faults), old unused pumps and intentionally damaging a small number of pumps. In the end, we obtained a very diverse dataset that contained a combination of real customer data and some intentionally damaged pumps from test benches.

At the hardware level, developing a system that reliably collected data 24/7 with minimal downtime is a challenging task. The hardware can be down, collect incorrect data or produce noisy readings. A system that works on a lab bench may become unreliable on site, and troubleshooting hardware and firmware issues can be time and resource consuming. From the design point of view, the hardware platform selection is determined by the cost, reliability and machine learning algorithm performance, which, in turn, requires a sufficient amount of data to be collected to design and tune correctly, resulting in a circular problem. This, to some extent, has been addressed in our project by an architectural decision of using lightweight IoT devices for collecting raw data and performing all processing, including machine learning and data storage remotely on the server. In the future, it may be interesting to experiment with distributed processing, where the fault detection is made locally by the device to minimise the reliance on the centralised server and connectivity problems.

Finally, at the machine learning level, the limited dataset size and the fact that data are unbalanced towards the non-faulty signatures may lead to bias and overfitting. Therefore, it is important to test the model under diverse scenarios, ensuring the model generalises well for new data. The only reliable way to achieve this is testing the model with more data, which requires maintaining and operating the sensing infrastructure for an extended period of time, long enough for a sufficient number of pumps to fail to obtain the faulty signatures. Considering the relatively long lifespan of modern pumps, this may require several more years of data collection across several sites. This presents an immense practical and financial difficulty and is perhaps the main reason behind the scarcity of prior work involving real pump data. The contribution of this study is the development of such an infrastructure and offering an insight into the ML performance with real rather than simulated data.

Furthermore, given that the non-faulty pump/motor can become faulty over-time, the data that are used to train the model needs to be regularly checked to maintain the correctness. We also realized that the way the DQ images are resized, center cropped, etc., or the format they were saved/plotted vastly affected the model’s performance. Therefore, it needs to be noted that the training on saved images vs. detecting on images on buffer needs to be configured as equally as possible.

## 6. Conclusions and Future Work

In conclusion, this paper presents a novel approach for centrifugal pump induction motor fault detection using data from real operational pumps deployed at various sites. This study reviewed the state-of-the-art residual network models on Concordia patterns and proposed an algorithm based on the image-based analysis of DQ patterns, which showed a promising 85.51% accuracy. The authors believe this is the first study that used a combination of transfer learning and DQ transformation to classify motor faults and a rare research paper that is based on real customer data. It is hoped that this study will provide valuable insights to other researchers on experimenting with image detection techniques on motor fault detection.

Potential future work includes expanding the training, validation and test datasets with data from more pumps, as well as predicting multiple fault possibilities instead of using a binary classification. Although the method was developed for centrifugal pumps, it would be interesting to explore the potential for other applications where alternate current (AC) at a known frequency is used to power electric motors. The DQ representation of the electric current signatures could be used to detect potential failures. Finally, the fault classification was performed in batch mode in this study. Processing the data in real-time and making classification online as samples arrive from the sensors may be beneficial in terms of latency and is potential future work.

## Figures and Tables

**Table 1:** Training pump specifications.

ID	Fault	Voltage (V)	Speed (rpm)
Tr_F1	Stator winding	415	1385
Tr_F2	Stator winding	415	1385
Tr_F3	Excess vibr., bearing dmg.	400	2815
Tr_NF1	Non-faulty	415	1450
Tr_NF2	Non-faulty	415	1450
Tr_NF3	Non-faulty	400	1450

**Table 2:** Validation pump specifications.

ID	Fault Type	Voltage (V)	Speed (rpm)
Va_F1	Impeller dmg., excess vibr.	400	1420
Va_NF1	Non-faulty	400	1450
Va_NF2	Non-faulty	400	1450
Va_NF3	Non-faulty	400	2815
Va_NF4	Non-faulty	415	1445
Va_NF5	Non-faulty	415	1445

**Table 3:** Testing pump specifications.

ID	Fault Type	Pump Type	Voltage (V)	Speed (rpm)
Te_F	Cavitation	Booster pump	425	2800
Te_NF	Non-faulty	Booster pump	415	2860

**Table 4:** Dataset sizes for each sub-dataset where training dataset was augmented.

Dataset Name	Faulty Data	Non-Faulty Data	Total
Training	6348	5688	12,036
Validation	370	538	908
Testing	107	107	214

**Table 5:** Precision, recall and accuracy tables for validation and testing datasets.

Validation				
**\**	**Precision (%)**	**Recall (%)**	**F1-Score**	**Accuracy (%)**
Non-Faulty	87	95	91	88.33
Faulty	91	79	85	
**Testing**				
Non-Faulty	94	76	84	85.51
Faulty	80	95	87	

**Table 6:** Testing dataset size and results for each sampling rate and pump for ResNet-34.

Sampling Frequency (Hz) Correct Labels out of All Tests				
Unseen Pump ID	1500	3000	4500	Accu/Pump (%)
Faulty Pump	35/37	17/20	50/50	95.33
Non-Faulty Pump	20/37	11 / 20	50/50	75.70
Accu/Samp. Rate	74.32	70.00	100.00	85.51

**Table 7:** Confusion matrices for 1500 Hz, 3000 Hz, 4500 Hz and the overall data.

1500 Hz	Faulty (Actual)	Non-Faulty (Actual)	3000 Hz	Faulty (Actual)	Non-Faulty (Actual)	4500 Hz	Faulty (Actual)	Non-Faulty (Actual)	Overall	Faulty (Actual)	Non-Faulty (Actual)
Faulty (Predicted)	35 (94.59%)	17 (45.95%)	Faulty (Predicted)	17 (85%)	9 (45%)	Faulty (Predicted)	50 (100%)	0 (0%)	Faulty (Predicted)	102 (95.33%)	26 (24.30%)
Non-Faulty (Predicted)	2 (5.41%)	20 (54.05%)	Non-Faulty (Predicted)	3 (15%)	11 (55%)	Non-Faulty (Predicted)	0 (0%)	50 (100%)	Non-Faulty (Predicted)	5 (4.67%)	81 (75.70%)

## Data Availability

The data collected and analysed in this study belongs to Deckpro Pumps/Uptime Systems Ltd. and can only be shared with their consent.
